# Méga-œsophage révélé par une pleurésie

**DOI:** 10.11604/pamj.2017.28.181.13721

**Published:** 2017-10-27

**Authors:** Amadou Diop Dia, Samba Niang

**Affiliations:** 1 UFR des Sciences de la Santé, Université Gaston Berger de Saint-Louis, Sénégal

**Keywords:** Méga-œsophage, pleurésie, gastro-œsophagien, Megaesophagus, pleurisy, gastro- esophageal

## Abstract

We report the case of a 65-year old patient without a history of alcohol consumption and cigarette smoking but with a history of gastrœsophageal reflux disease for which he had never been examined. He was admitted to the Department of Pneumology for exploration of a dry hacking cough causing insomnia associated with diffuse heaviness in his right chest evolving over a month in a context of evening and night fever without weight loss. Clinical and radiological examination showed right pleural effusion syndrome. Radiographic follow-up after evacuating pleural effusion by puncture showed inhomogeneous opacity occupying nearly all the right lung (A). Chest CT scan objectified thoracic megaesophagus associated with non-specific pleuro-pulmonary disease. The patient underwent upper gastrointestinal endoscopy that didn’t show suspect cancer signs. Pleuro-pulmonary disease outcome was favorable after amoxicillin treatment.

## Image en médecine

Il s'agit d'un homme de 65 ans, non alcoolo-tabagique, aux antécédents médicaux de reflux gastro-œsophagien pour lequel il n'a jamais consulté. Il est admis au service de pneumologie pour l'exploration d'une toux quinteuse sèche, insomniante, associée à une douleur thoracique droite diffuse à type de pesanteur évoluant depuis 1 mois environ dans un contexte de fièvre vespéro-nocturne sans perte de poids. Les examens clinique et radiologique avaient retrouvé un syndrome d'épanchement liquidien pleural droit. La radiographie de contrôle réalisée après la ponction pleurale évacuatrice, avait mis en évidence une opacité inhomogène occupant la quasi-totalité du champ pulmonaire droit (A). Le scanner thoracique était en faveur d'un mégaœsophage thoracique associé à une pleuro-pneumopathie non spécifique (B). Le patient a bénéficié d'une endoscopie digestive haute qui n'avait pas retrouvé de signe suspect de cancer. L'évolution de la pleuro-pneumopathie était favorable après un traitement par amoxicilline

**Figure 1:**
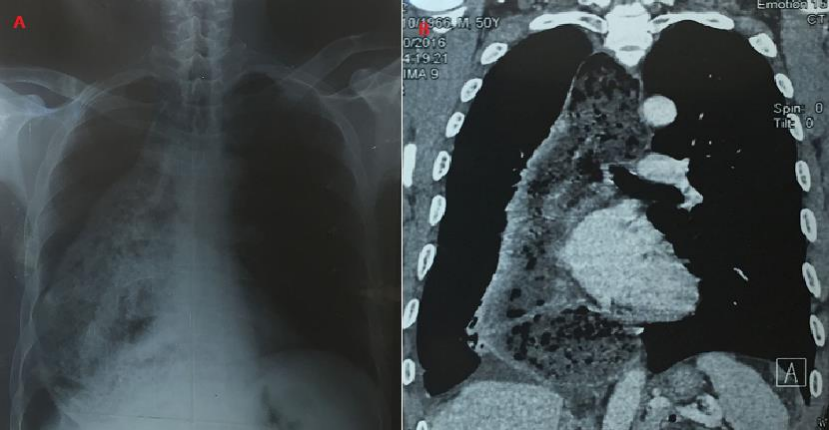
(A) radiographie thoracique de face: opacité inhomogène occupant les lobes moyen et inférieur du poumon droit; (B) scanner thoracique: méga-œsophage thoracique mesurant 78 mm de diamètre associée à une pleuropneumopathie droite

